# The IL-33 Receptor ST2 Regulates Pulmonary Inflammation and Fibrosis to Bleomycin

**DOI:** 10.3389/fimmu.2018.01476

**Published:** 2018-06-25

**Authors:** Manoussa Fanny, Mégane Nascimento, Ludivine Baron, Corinne Schricke, Isabelle Maillet, Myriam Akbal, Nicolas Riteau, Marc Le Bert, Valérie Quesniaux, Bernhard Ryffel, Aurélie Gombault, Sandra Même, William Même, Isabelle Couillin

**Affiliations:** ^1^University of Orleans and CNRS, UMR7355, Orleans, France; ^2^University of Orleans and CNRS, UPR4301, Orleans, France

**Keywords:** bleomycin, lung, inflammation, fibrosis, suppression of tumorigenicity 2, interleukin-33, magnetic resonance imaging

## Abstract

Idiopathic pulmonary fibrosis is a progressive, devastating, and yet untreatable fibrotic disease of unknown origin. Interleukin-33 (IL-33), an IL-1 family member acts as an alarmin with pro-inflammatory properties when released after stress or cell death. Here, we investigated the role of IL-33 in the bleomycin (BLM)-induced inflammation and fibrosis model using mice IL-33 receptor [chain suppression of tumorigenicity 2 (ST2)] mice compared with C57BL/6 wild-type mice. Unexpectedly, 24 h post-BLM treatment ST2-deficient mice displayed augmented inflammatory cell recruitment, in particular by neutrophils, together with enhanced levels of chemokines and remodeling factors in the bronchoalveolar space and/or the lungs. At 11 days, lung remodeling and fibrosis were decreased with reduced M2 macrophages in the lung associated with M2-like cytokine profile in ST2-deficient mice, while lung cellular inflammation was decreased but with fluid retention (edema) increased. *In vivo* magnetic resonance imaging (MRI) analysis demonstrates a rapid development of edema detectable at day 7, which was increased in the absence of ST2. Our results demonstrate that acute neutrophilic pulmonary inflammation leads to the development of an IL-33/ST2-dependent lung fibrosis associated with the production of M2-like polarization. In addition, non-invasive MRI revealed enhanced inflammation with lung edema during the development of pulmonary inflammation and fibrosis in absence of ST2.

## Introduction

Idiopathic pulmonary fibrosis (IPF) is a progressive, chronic, irreversible, and lethal lung disease. The disease process is initiated through alveolar epithelial cell microinjuries that lead to a persistent immuno-inflammatory phase with production of cytokines, chemokines, and growth factors responsible for the expansion of fibroblast and myofibroblast populations (1). The expansion of fibroblasts and myofibroblasts leads to the dysregulation of tissue repair, which induces destruction of the parenchyma (2–4). While the etiology is not fully understood, it has become clear that many respiratory diseases are promoted, at least initially by a strong inflammatory response (1). Studies delineating the precise role of inflammation and immunity are needed to better characterize the mechanisms involved. We previously showed that bleomycin (BLM) induces uric acid and ATP release that act as a danger signals, ATP acting through its receptor P2X7, both leading to NLR pyrin domain containing 3 inflammasome-dependent IL-1β secretion and lung fibrosis (5–8). However, the role of IL-1 family member interleukin-33 (IL-33) and its receptor suppression of tumorigenicity 2 (ST2) in pulmonary inflammation and fibrosis is unclear. IL-33 acts as a dual-function protein, with both nuclear and extracellular effects when released as a danger signal upon cellular damage (9, 10). IL-33 is constitutively expressed in the nucleus of endothelial and/or epithelial cells where it associates with chromatin (11–14) and is involved in maintaining barriers (15). Extracellular IL-33 interacts with the ST2 receptor which is either expressed on the cell surface (membrane-bound ST2L) or shed from these cells [soluble ST2 (sST2)], thereby functioning as a “decoy” receptor to bind and efficiently inhibit IL-33 activity (16). ST2L is closely related to the IL-1 receptor 1 (IL-1R1) and binding of IL-33 on ST2L activates NF-κB pathway (17), suggesting that it regulates the response (16, 18). IL-33 is produced as a precursor or in full-length form (266 amino acids in mice) with the typical IL-1-like cytokine domain localized at the C terminal (19). Unlike IL-1β, full-length IL-33 is bioactive and may be processed by serine proteases secreted by activated neutrophils, generating 20- to 30-fold more active forms (20).

ST2 expression was shown to be increased in the mouse model after BLM administration or in patients upon acute exacerbation of pulmonary fibrosis (21, 22). Moreover, recent studies showed that IL-33 potentiates BLM-induced lung injury (23). Another study showed that a treatment with a lentivirus expressing sST2 improved survival rate, reduced weight loss, and profoundly attenuated pulmonary inflammatory cell infiltration, fibrotic changes, and levels of IL-33 and TGF-β1 levels in the airways after BLM (24). By contrast, following transient ST2 overexpression before BLM administration, ST2 was shown to dampen the initial stage of acute lung injury (25) and to promote lung fibrosis in a ST2-dependent manner through the induction of alternatively activated macrophages and innate lymphoid cells (26) while sST2 suppressed the initial stage of BLM-induced lung injury (25). The increased concentration of sST2 in serum may be a biomarker of IPF (21). Full-length IL-33 may be pro-inflammatory and pro-fibrotic effects through its intracellular form, IL-33 remaining predominantly intracellular (27). However, the role of IL-33 and IL-33/ST2 signaling in establishment of pulmonary inflammation and fibrosis is not well understood. The IL-33/ST2 axis was shown to have an anti-inflammatory effect (26).

Here, we revisited the role of the IL-33/ST2 in the BLM model of pulmonary inflammation and fibrosis by both classical immunologic methods and magnetic resonance imaging (MRI). The results indicate that MRI provides important information to monitor the evolution of edema in the mouse lung. We show that the absence of ST2 results in increased early inflammation with fluid retention, but decreased fibrosis. Our data reveal that the IL-33 pathway leads to shift from acute pulmonary inflammation and remodeling induced by lung damage to an excessive lung repair response with fibrosis through the production of M2-like polarization. Regulation of this IL-33/ST2 axis may attenuate pulmonary fibrosis and enhance recovery. In addition, we show that MRI allows a rapid non-invasive detection of lung edema during the development of pulmonary fibrosis.

## Results

### ST2-Deficient Mice Display Exacerbated Airway Inflammation to BLM 24 h After BLM Exposure

**Figure 1:**
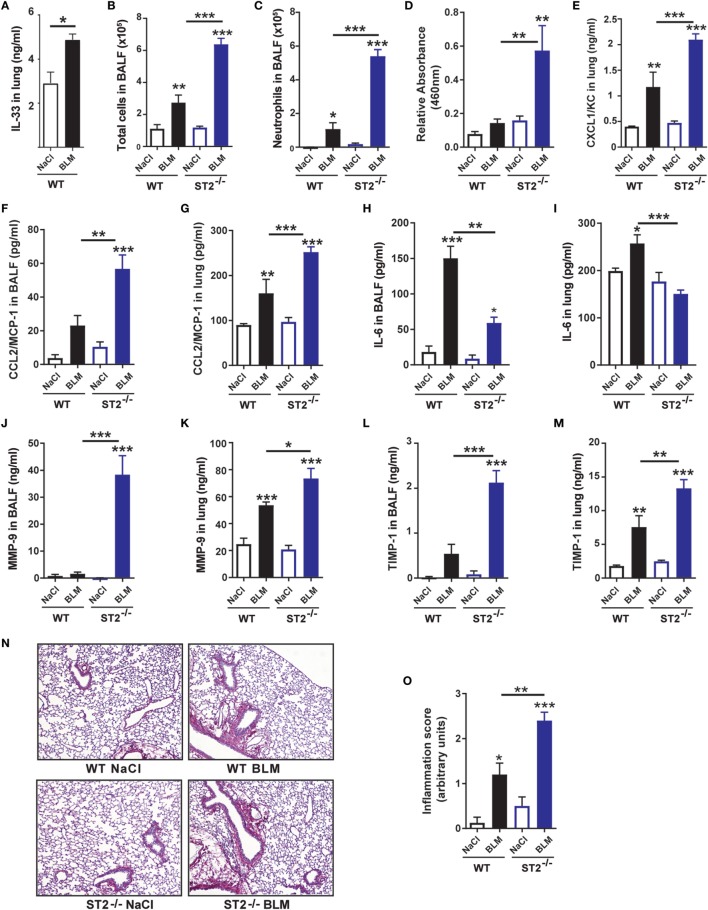
ST2-deficient mice display an exacerbated inflammatory response to bleomycin (BLM) 24 h after exposure. Wild-type (WT) mice and ST2-deficient mice (ST2^−/−^) were instilled with 7.5 mg/kg of BLM or saline and inflammation parameters were assayed at day 1. Interleukin-33 (IL-33) contents in the lungs **(A)**, total cell **(B)**, and neutrophil **(C)** numbers in the bronchoalveolar lavage fluid (BALF) were significantly increased in BLM-treated ST2^−/−^ when compared with WT mice. Cell influx was in correlation with increased lung myeloperoxidase activity **(D)** and enhanced levels the neutrophil chemoattractant chemokine, CXCL1/KC in lung **(E)**, of the monocyte chemoattractant CCL2/MCP-1 levels in BALF **(F)** and lung **(G)** and the interleukin-6 (IL-6) cytokine in BALF **(H)** and lung **(I)**. The tissue remodeling factors matrix metalloproteinase (MMP)-9 and tissue inhibitor of metalloproteinase (TIMP)-1 were higher in BALF and lung **(J–M)**. Histological lung sections of 5-µm were stained with picrosirius red (original magnification 200×). Histological micrographs showed increased parenchyma infiltrating cells **(N)** and increased inflammation score **(O)** after BLM instillation. Data are representative of three independent experiments and are expressed as mean values ± SEM (*n* = 4–6 mice per group, *p < 0.05, **p < 0.01, ***p < 0.001).

We observed that IL-33 was expressed in lung homogenates of naïve C57BL/6 wild-type (WT) mice and was significantly increased after BLM instillation (Figure 1A) whereas IL-33 remained undetectable in bronchoalveolar lavage fluid (BALF) (data not shown). In order to decipher the role of the IL-33/ST2 axis in the pulmonary inflammation induced by BLM, we performed saline or BLM intranasal instillation in WT and ST2-deficient (ST2^−/−^) mice. Lung inflammation was characterized by an increased recruitment of total cells (Figure 1B), primarily neutrophils (Figure 1C) in the airway of BLM-treated WT mice, which were drastically augmented in BLM-treated ST2^−/−^ mice. Exacerbated neutrophil recruitment in ST2^−/−^ mice was associated with increased myeloperoxidase (MPO) activity (Figure 1D) and neutrophilic chemokine CXCL1/KC levels in the lungs (Figure 1E). In addition, monocyte chemokine CCL2/MCP-1 levels were higher in the BALF and the lungs of BLM-treated ST2^−/−^ mice (Figures 1F,G). By contrast, levels of the interleukin-6 (IL-6) known to induce the expression of the IL-4 receptor on macrophages (28) were decreased in the BALF and the lungs of BLM-treated ST2^−/−^ mice (Figures 1H,I). Finally, expression of the remodeling factors matrix metalloproteinase (MMP)-9 (Figures 1J,K) and of the tissue inhibitor of metalloproteinase (TIMP)-1 (Figures 1L,M) upregulated by BLM in BALF and lung of WT mice were higher in ST2-deficient mice. In line with the above findings, lung histological analysis showed increased inflammatory cell recruitment into the lung in BLM ST2^−/−^ mice when compared with WT counterparts (Figures 1N,O).

### ST2-Deficient Mice Display No Difference of Total Cell, but Reduced Recruitment of Alternative Macrophage 11 Days After BLM Exposure

**Figure 2:**
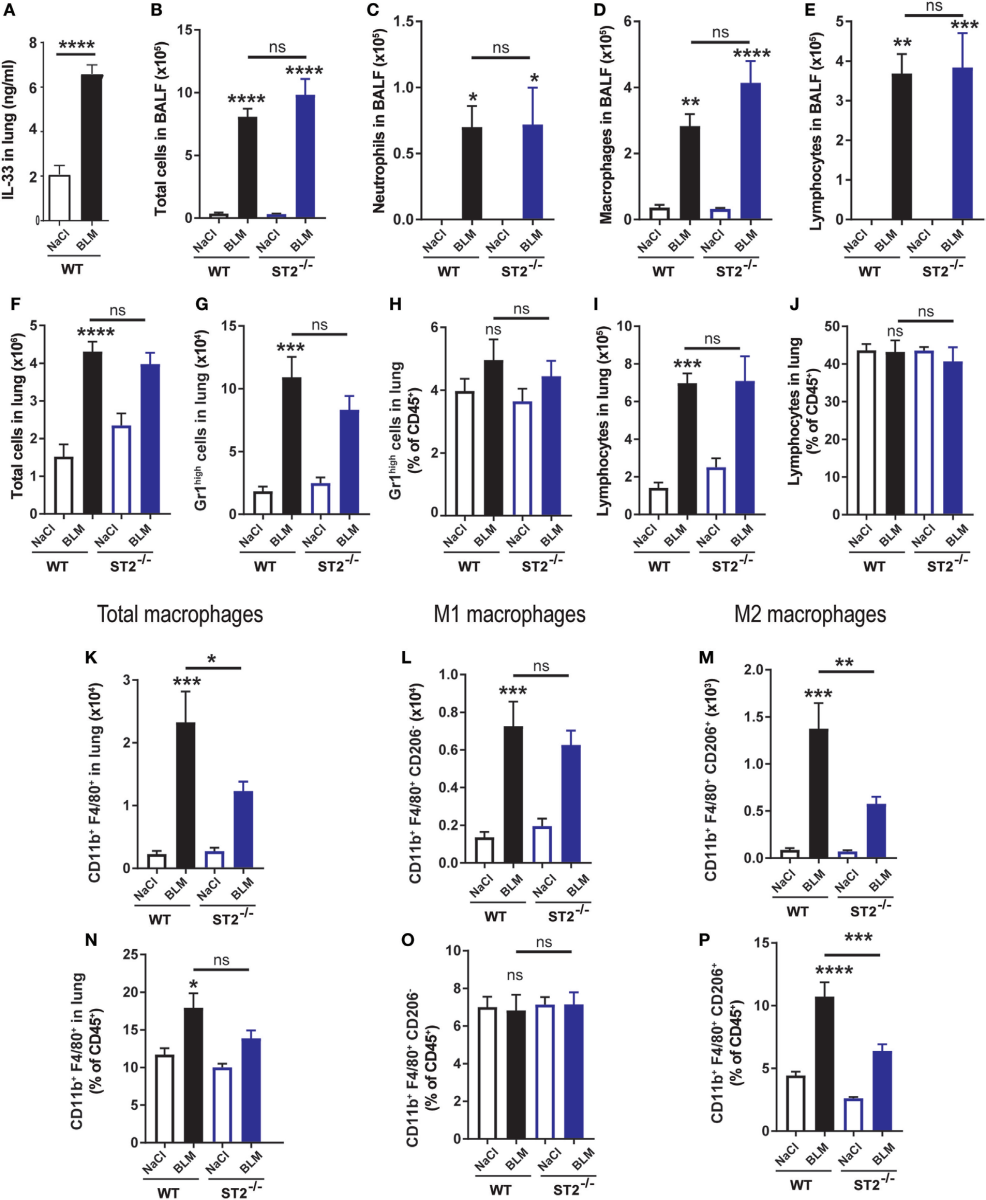
Reduced inflammation by alternative macrophage in ST2-deficient mice 11 days after bleomycin (BLM) exposure. Wild-type (WT) and ST2^−/−^ were instilled with of BLM (3 mg/kg) and harvested at day 11. Interleukin-33 (IL-33) contents in the lungs of WT **(A)**, count of total cells **(B)**, neutrophils **(C)**, macrophages **(D)**, and lymphocytes **(E)** in bronchoalveolar lavage fluid (BALF) were analyzed in NaCl or BLM-treated ST2^−/−^ mice in comparison to WT mice. Flow cytometry analysis of cells recruited into the lung parenchyma was performed for NaCl- or BLM-treated WT or ST2-deficient mice. Total cell number **(F)**, neutrophils **(G,H)**, and lymphocyte **(I,J)** number or percentage were presented. Data are representative of three independent experiments. In addition, analysis of lung macrophage subsets was performed and number or percentage of CD11b^+^F4/80^+^ macrophages **(K,N)**, CD11b^+^F4/80^+^ CD206^−^ classical (M1) macrophages **(L,O)** or CD11b^+^F4/80^+^ CD206^+^ alternative (M2) macrophages **(M,P)** were shown. Experiments are expressed as mean values ± SEM (*n* = 4–6 mice per group, *p < 0.05, **p < 0.01, ***p < 0.001).

To investigate the role of the IL-33 receptor ST2 in chronic inflammation and fibrosis, WT and ST2^−/−^ mice were instillated with BLM (3 mg/kg) or NaCl as control and inflammation was analyzed 11 days post-BLM treatments. Lung IL-33 levels remained elevated in BLM-instillated WT mice in comparison to NaCl mice (Figure 2A) and were slightly higher when compared with day 1 post-BLM (Figure 1A), but were not detected in BALF (data not shown). When compared with WT mice, BLM-treated ST2^−/−^ mice displayed a trend, but not significant increase of total cell (Figure 2B), neutrophils (Figure 2C), macrophages (Figure 2D), and lymphocytes (Figure 2E) recruited into the BALF. In addition, using flow cytometry analysis (FACS) of cells recruited into the lung parenchyma, we observed not difference in total cell (Figure 2F), neutrophil (Figures 2G,H), and lymphocyte (Figures 2I,J) numbers and percentages between WT and ST2^−/−^ mice. By contrast, we report a significant decreased in the number of CD11b^+^FA/80^+^ lung macrophage but only a trend of decreased frequency (Figures 2K,N). While no difference in the number and frequency of CD11b^+^F4/80^+^ CD206^−^ classical (M1) macrophages (Figures 2L,O) was recorded, a significant reduction in the number and percentage of CD11b^+^F4/80^+^ CD206^+^ alternative (M2) macrophages (Figures 2M,P) was found in lungs of ST2-deficient mice. These results suggest that BLM induces a M1 to M2 polarization *in vivo*, which depends on IL-33/ST2 signaling.

### ST2-Deficient Mice Display Decreased Expression of M2 Macrophage Mediators

**Figure 3:**
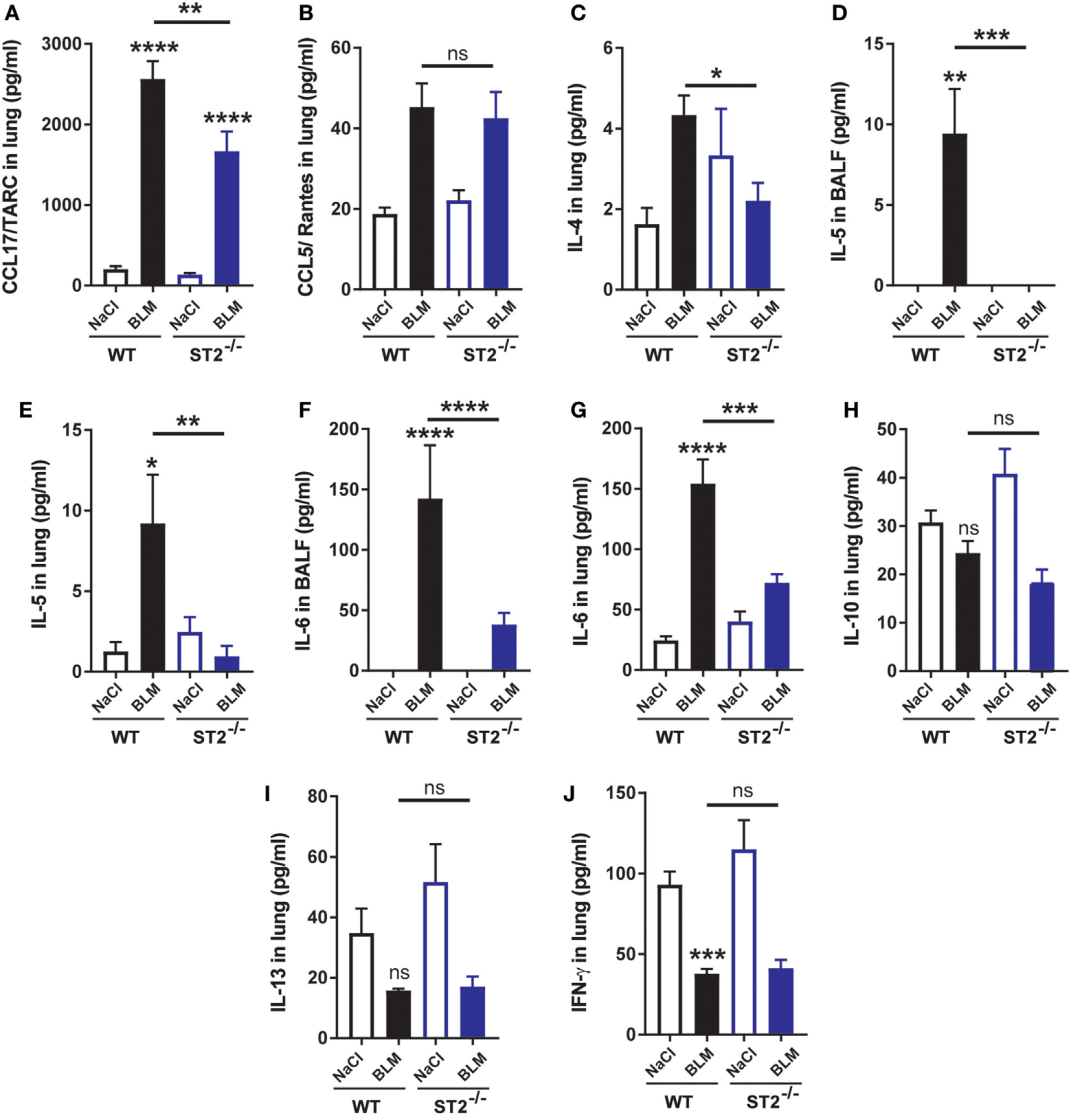
Decreased expression of M2 macrophage-associated mediators in ST2-deficient mice. Expression in lung of the chemokine CCL17/TARC in lungs was measured by ELISA **(A)**. Data are representative of three independent experiments. In addition expression in lung of the chemokine CCL5/RANTES **(B)** and of the cytokines IL-4 in lung **(C)** and IL-5 in bronchoalveolar lavage fluid (BALF) **(D)** and IL-5 **(E)**, interleukin-6 (IL-6) in BALF **(F)** and lung **(G)**, IL-10 **(H)**, IL-13 **(I)**, and IFN-γ **(J)** in lungs were analyzed by multiplex immunoassay (Bio-Rad). Experiments are expressed as mean values ± SEM (*n* = 4–6 mice per group, *p < 0.05, **p < 0.01, ***p < 0.001, ****p < 0.0001).

We then investigated expression of inflammatory mediators. Analyzing expression of the CCL17/TARC chemokine characteristic M2 macrophage profile, we report decreased levels of the CCL17/TARC chemokine in lung of ST2-deficient mice (Figure 3A) in contrast to unchanged levels of the chemokine CCL5/RANTES (Figure 3B). Moreover, performing multiplex analysis, we analyzed the expression of the characteristic M2 cytokines IL-4 and IL-5. BLM airway instillation induced IL-4 expression in lung (Figure 3C) and IL-5 expression in BALF and lung (Figures 3D,E) in WT mice. We observed significant reduction of IL-4 levels in lung (Figure 3C) and IL-5 levels in BALF and lung (Figures 3D,E) in ST2^−/−^ mice indicating that production of cytokines representative of alternative M2 macrophages and T helper type 2 (Th2) cells depends on ST2 signaling. In addition, we report reduction of the M2 polarizing cytokine IL-6 in BALF and lung (Figures 3F,G) in ST2^−/−^ mice in comparison to WT mice in response to BLM. On the other hand, we observed no effect of BLM instillation on the expression of the anti-inflammatory cytokine IL-10 upon airway BLM in lung of WT or ST2^−/−^ mice (Figure 3H). In addition, the expression of the IL-13 cytokine known to be produced by innate lymphoid cell type 2 (ILC2) and Th2 cells, but not by M2 macrophages, was not significantly changed by BLM and between WT and ST2^−/−^ mice (Figure 3I). Finally, the expression of the T helper1 (Th1)-like cytokine IFN-γ in lung was reduce after BLM instillation in both WT and ST2^−/−^ mice (Figure 3J). These results indicates that the IL-33/ST2 pathway leads to a shift from M1 to M2 macrophage differentiation and suggest that M2 macrophages are the main immune cells involved in inflammation resolution and promoting tissue repair.

### Reduced Pulmonary Fibrosis in ST2-Deficient Mice 11 Days After BLM Exposure

**Figure 4:**
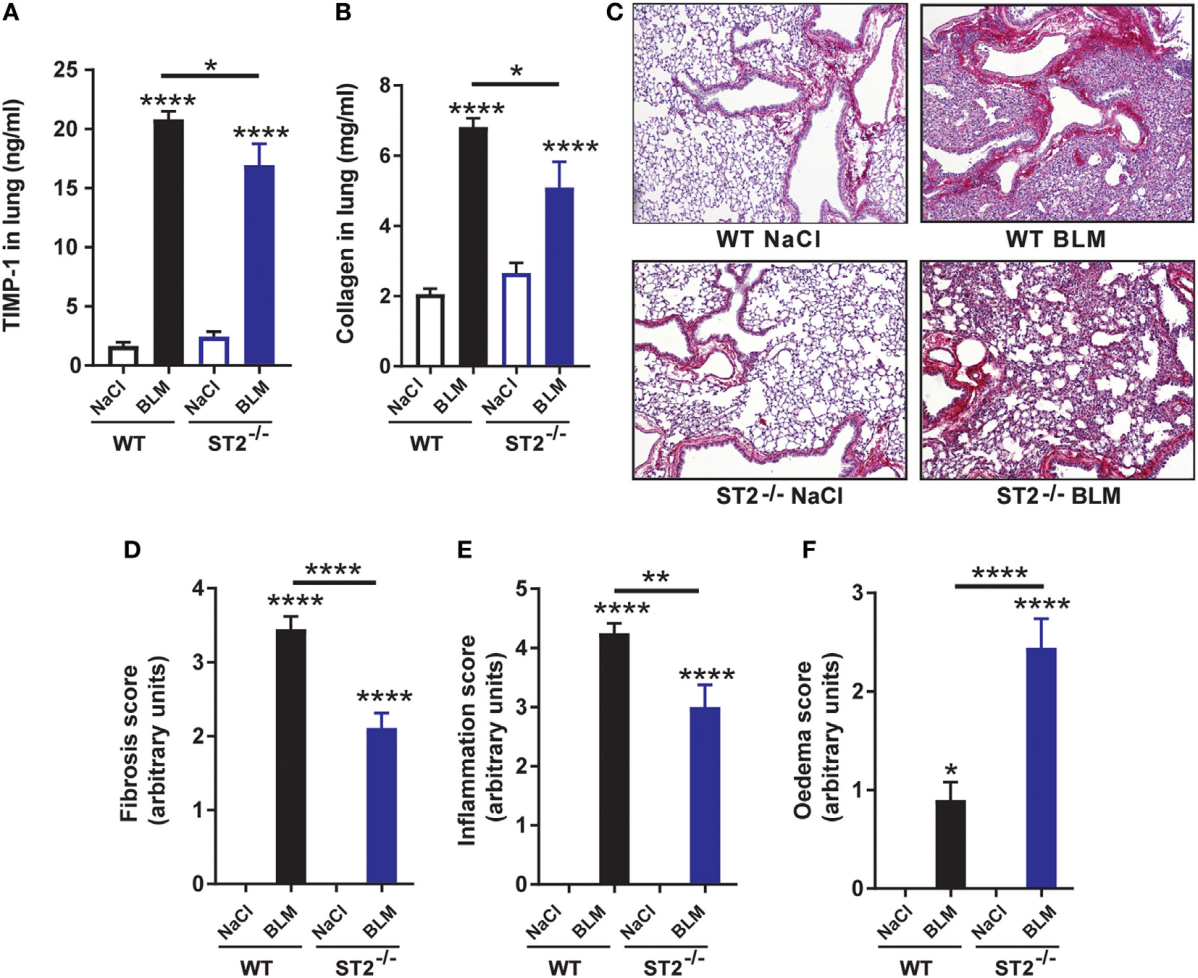
Reduced pulmonary fibrosis in ST2-deficient mice at day 11 after bleomycin (BLM) instillation. Tissue inhibitor of metalloproteinase (TIMP)-1 remodeling factor levels **(A)** and total collagen content **(B)** in lungs were measured by ELISA and Sircol assay, respectively. Histological lung sections of 5-µm were stained with picrosirius red (original magnification 200×). Histological micrograph of representative lung sections **(C)** and semi-quantitative severity scores of fibrosis **(D)**, cellular inflammation **(E)**, or edema **(F)** were shown. All experiments are expressed as mean values ± SEM (*n* = 4–6 mice per group, *p < 0.05, **p < 0.01, ****p < 0.0001).

Interestingly, we observed reduced body weight loss in ST2^−/−^ mice in comparison to WT mice (data not shown). Moreover, the level of the remodeling factor TIMP-1, a marker of evolution toward fibrosis, was significantly lower in lung of ST2^−/−^ mice in comparison to WT mice at day 11 (Figure 4A). In addition, total collagen content in the lungs (Figure 4B) was lower in ST2^−/−^ mice. Finally, histological analysis revealed a significant reduction of lung fibrosis in the absence of ST2 as shown by representative lung sections and semi-quantitative severity scores (Figures 4C,D) and inflammation score (Figure 4E). Moreover, inflammation in the airways was associated with enhanced extracellular fluid retention expressed as edema upon BLM instillation which was increased in ST2-deficient mice (Figure 4F). These results suggest that ST2 deficiency impairs the development of lung fibrosis, but enhances edema in response to BLM.

### Augmented Extracellular Fluid Retention in the Airways Assessed by MRI

**Figure 5:**
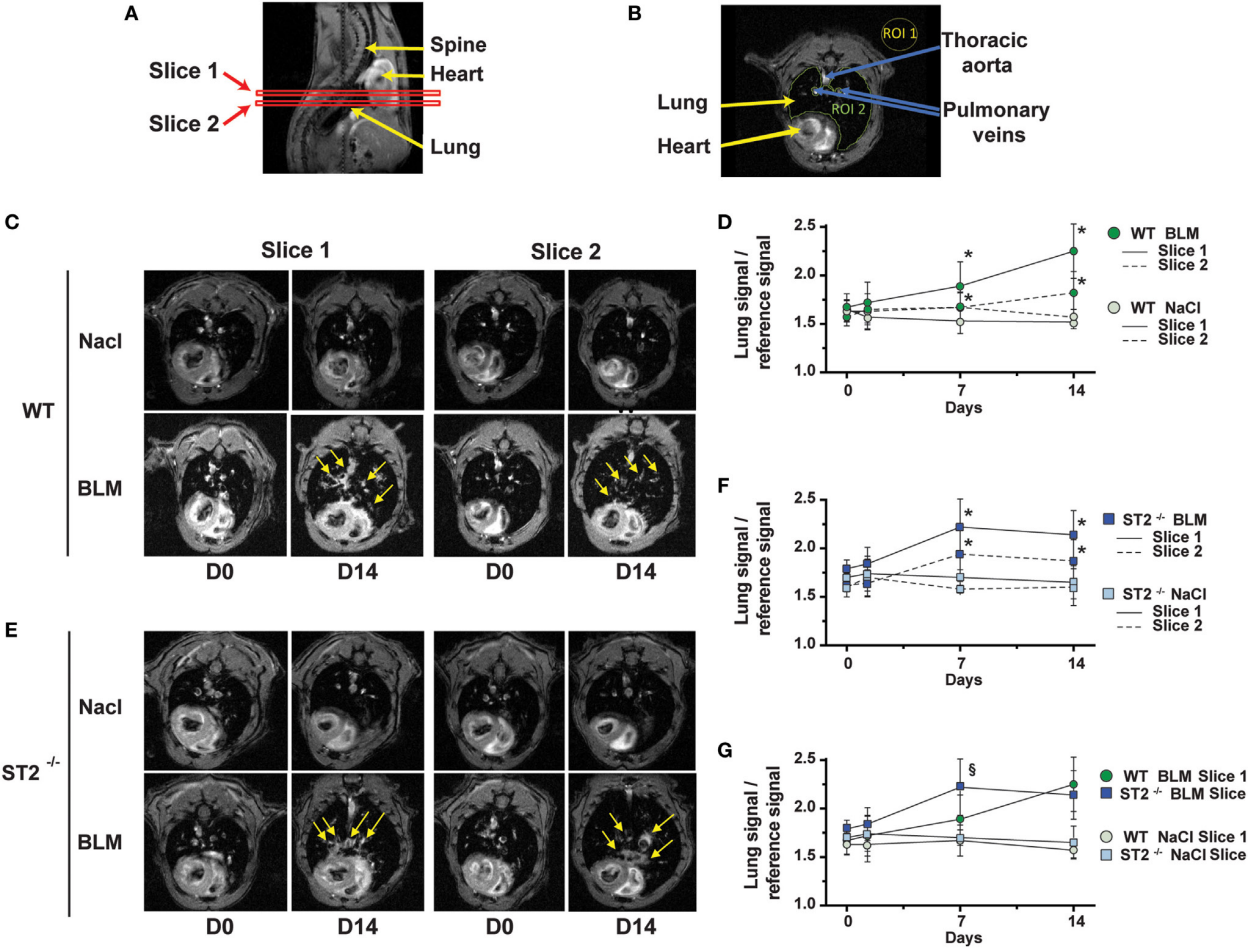
Augmented edema in airways in absence of ST2 after bleomycin (BLM) instillation. Magnetic resonance imaging (MRI) sagittal scan of the mouse with the position the two MRI slices through the lung cavity **(A)**. Axial MRI scans of mouse lungs **(B)**. The image shows the two selected region of interests (ROIs) used to calculate the signal to noise ratio: the ROI 1 (yellow), corresponding to the reference signal and the ROI 2 (green) of the signal of the lungs. Transverse (axial) thoracic MRI from wild-type (WT) **(C)** or ST2^−/−^ mice **(E)** before (D0) and 2 weeks (D14) following single BLM administration. The arrows indicate the fluid signal detected by MRI at the different time point. Signal to noise ratio (lung signal in ROI 2/reference signal in ROI1) at days 0, 1, 7, and 14 before and after instillation with NaCl (light green circles) or BLM (dark green circles), calculated from the MR images from slice 1 (full line) and slice 2 (dotted line) in WT mice **(D)**. Signal to noise ratio at days 0, 1, 7, and 14 before and after instillation with NaCl (light blue squares) or BLM (dark blue squares), calculated from the MR images from slice 1 (full line) and slice 2 (dotted line) in ST2^−/−^ mice **(F)**. Comparison of the signal to noise ratio in slice 1, at days 0, 1, 7, and 14 before and after instillation with NaCl (light colors) or BLM (dark colors) in WT mice (circles) and ST2^−/−^ mice (squares) **(G)**. Data are representative of two independent experiments and are expressed as mean values ± SEM [*n* = 4–6 mice per group, (*) (*p* < 0.05), (§) (*p* < 0.05)].

Non-invasive *in vivo* imaging by MRI was used to evaluate the effects of BLM instillation on fluid retention and inflammation in WT and ST2^−/−^ mice. MRI recording were performed at two anatomical levels, corresponding to slices 1 and 2 as shown in Figure 5A which represent axial slices through the chest of allowed to assess the structure of the lung and neighboring organs (Figure 5B). Typical MRI images of healthy lungs appeared predominantly dark because of the low signal associated with air space of the lung parenchyma. Importantly, MRI baseline measurements before BLM administration (day 0) allowed the use of each animal as its own control. There was no significant difference in signal intensity in the lungs following saline instillation at any time point throughout the duration of the experiment (Figures 5C,G), and no significant changes of MRI parameters were detected 24 h after BLM instillation in WT and ST2^−/−^ mice (data not shown). However, representative axial sections revealed a significant increase of MRI fluid signal at day 7 and 14 in WT (Figure 5D) and ST2^−/−^ mice (Figure 5F). BLM-induced edema was apparent around smaller secondary and tertiary bronchi (slice 2) known as bronchioles, but was more pronounced around larger bronchi (slice 1) indicating inflammation of upper airways (large airways) rather than lower airways (small airways) at these stages (Figure 5C). In absence of ST2, a prominent the MRI signal was also found in the bronchi and bronchioles. This edema is characterized by augmentation of the MRI signal (in white, as indicated by yellow arrows) (Figure 5E). This difference of signals was quantified as shown in graphs (Figures 5F,G). The signal peaked at day 7 (slice 1 and slice 2) and remained significantly elevated until day 14 in ST2^−/−^ mice. Importantly, the MRI signal in the lung was significantly increased in ST2^−/−^ mice in comparison to WT mice, suggesting an earlier edema in ST2^−/−^ mice (Figures 5E,G). These results suggest that ST2^−/−^ mice present more severe edema in the inflamed airways when compared with WT mice in accordance with the microscopic data.

## Discussion

We reported before that BLM induces pulmonary inflammation through the inflammasome-dependent release of IL-1β expression and IL-1R1 signaling (5–8). Here, we focused on the controversial role of another IL-1 cytokine family member IL-33 in BLM-induced lung pathology. Using a clinically approved BLM as a reliable source of the drug, we observed that BLM enhanced the expression of IL-33 during acute inflammation, which remained elevated during chronic inflammation. Moreover, we show for the first time that ST2 deficiency leads to acute exacerbated pulmonary inflammation. Neutrophil influx was associated with enhanced expression of the chemokines CXCL1 and CCL2 and of the remodeling factors MMP-9 and TIMP-1 in the bronchoalveolar space and lung parenchyma, and increased cellular inflammation in lung parenchyma, 24 h after BLM airway instillation. By contrast, the expression of the cytokine IL-6 commonly associated with pro-inflammatory functions was decreased in absence of ST2. Interestingly, an *in vitro* study reported recently that IL-6 enhances the polarization of alternatively activated macrophages through the upregulation of the IL-4Rα chain of the IL-4 receptor and independently of IL-10 (28). Importantly, our results show that IL-6, which may favor a shift of pro-inflammatory classical macrophages (M1) into anti-inflammatory pro-fibrotic alternative macrophages (M2), is produced very early (day 1 after BLM) and dependent on IL-33/ST2 signaling.

Lung macrophages are important innate immune cells associated with two major distinct phenotypes, a pro-inflammatory subset (or classically activated macrophages) with production of pro-inflammatory cytokine, and an anti-inflammatory subset (or alternatively activated macrophages) linked with wound healing and tissue repair processes (29). At day 11, we observed no difference between WT and ST2-deficient mice, in the number and frequency of total cells, neutrophils, lymphocytes, and macrophages infiltrating the airways or lung tissue. Nevertheless, analysis of the infiltrating macrophages revealed reduced numbers and frequency of alternative activated (M2) macrophages in ST2-deficient mice. This was associated with a decreased of M2 macrophages-associated mediators in ST2-deficient mice with reduced expression of the CCL17/TARC chemokine characteristic of M2 macrophage profile, IL-4 and IL-5 cytokines produced by M2 macrophages, and IL-6 cytokine, which was shown to be involved in polarizing the innate immune response toward M2 macrophage activation, 11 days after BLM exposure. Levels of the anti-inflammatory cytokine IL-10 did not change in accordance with an *in vitro* study showing that IL-6-induced M2 macrophage polarization was independent of IL-10 (28). The expression of the Th1-like cytokine IFN-γ which in association with IL-6 promote the production of IL-1β *in vitro* (28) was reduced after BLM instillation in both WT and ST2-deficient mice. Levels of IL-13 cytokine known to be produced by ILC2 and/or Th2 cells were not changed, unlike previously shown (26). Our results confirm that the IL-33/ST2 pathway leads to a shift from M1 to M2 macrophage polarization but suggest an important role for early production of IL-6 in lung and airways but no role for IL-10 and IL-13 at this stage in our model. In addition, we propose that pulmonary M2 macrophages are the most important cells responsible for lung fibrosis development in this model in comparison to ILC2 or type 2 helper T cells (Th2) producing IL-13, which seem dispensable for promoting inflammation resolution and tissue repair at this stage. Our results show that IL-6 is a pleiotropic cytokine involved in induction but also resolution of inflammation and occurrence of wound healing *in vivo*.

These responses were associated with TIMP-1 overexpression, collagen deposit, and pulmonary fibrosis, all being attenuated in ST2-deficient mice confirming the role of IL-33/ST2 pathway in tissue repair and wound scaring driving pulmonary fibrosis as reported before at days 7 and 14 (26). These authors reported reduced cellular inflammation in the bronchoalveolar space, in particular, decreased neutrophils and macrophages in ST2-deficient mice. In addition, they observed that IL-33 polarized M2 macrophages to produce IL-13 and induced the expansion of ILC2s to produce IL-13, whereas we report IL-4 and IL-5 but no IL-13 production suggesting a predominant role of M2 macrophages rather than ILC2 in enhancing profibrogenic cytokine production in an ST2- and macrophage-dependent manner and leading to lung fibrosis. The immune cell subsets involved in evolution to pulmonary fibrosis through the IL-33/ST2 pathway is still discussed with studies involving a role of ILC2 (26, 30), NK cells (31), and/or M2 macrophages (26, 32).

By contrast, we report increased extracellular fluid retention in ST2-deficient mice as evaluated by two different methods, histological quantification and *in vivo* MRI. MRI is a medical imaging technique used in diagnostic medicine and biomedical research to image internal organs and physiological processes in both health and disease. The use of this non-invasive MRI allows analyzing time-dependent development of acute and chronic inflammation *in vivo* in individual animals during disease progression. MRI analysis demonstrated a rapid development of fluid retention in the airways knows as edema upon BLM administration, which was detectable, but not significant after 1 day, but augmented at days 7 and 14. Importantly, the MRI signals were much stronger in the absence of ST2 at day 7 post-BLM, suggesting an increased fluid retention/edema.

Interleukin-33 activates various cell types by binding to its receptor complex consisting of ST2 and the IL-1 receptor accessory protein (IL-1RAcP). While we measured a sustained increase of IL-33 in the lung, we could not be detected IL-33 in the bronchoalveolar space. These results might be explained by an increasing availability of the IL-1RAcP chain for interaction with IL-1R1, enhancing pulmonary inflammation induced by IL-1α and IL-1β, which were also induced upon nanoparticle instillation (33). Another possibility is that intracellular IL-33 is involved in the inflammatory processes observed. Indeed, the functional role of nuclear IL-33 in myeloid cell remains poorly studied. Increased IL-33 expression was reported in lungs of patients with IPF disease, as well as in the BLM-induced lung injury in mice but most of the observed IL-33 expression was intracellular and intranuclear (27). IL-33 was shown to have pro-inflammatory and pro-fibrotic effects through its intracellular form, IL-33 remaining predominantly intracellular (27).

Using both classical immunologic methods and MRI to analyze inflammation, we observed enhanced early pulmonary inflammation. These results suggest that IL-33 through ST2 interaction promotes anti-inflammatory effects and has pro-fibrotic effect through membrane ST2, suggesting that inflammation resolution is necessary for the development of fibrosis. In addition, MRI allows a rapid, non-invasive detection of lung edema related to inflammation to monitor pulmonary inflammation and predict evolution to lung fibrosis. In addition, ST2 deficiency was associated with delayed resolution of fluid retention/edema, reduced shift from classical pro-inflammatory (M1) macrophages to alternative activated (M2) macrophages, which have anti-inflammatory and pro-fibrotic properties.

Our data are novel showing early prolonged, unresolved inflammation in absence of ST2 and that IL-33 is an important profibrogenic cytokine that signals through ST2 to promote the initiation and progression of pulmonary fibrosis essentially by recruiting IL-6-dependent alternative activated macrophages and directing pulmonary fibrosis. Even if it appears that M2 macrophages are involved in the aberrant wound-healing cascade during fibrosis, different subtypes were proposed corresponding to activating and produced cytokines (29). However, during the different stage of pulmonary fibrosis, macrophages may co-express markers of M1/M2 macrophage activation, showing that lung macrophages are highly plastic and may be representative of different activation states during lung fibrosis (34). However, it is not known whether the polarization of lung macrophages observed during pulmonary fibrosis is persistent or reflects transient activation states, or whether it is representative of fibrosis-specific functional heterogeneity. Better understanding of M2 macrophage kinetic and activation, and produced mediators may very important in order to identify new biomarkers and targets to treat pulmonary fibrosis.

## Materials and Methods

### Mice

8- to 14-week-old C57BL/6J WT mice were purchased from Janvier Labs (Le Genest Saint Isle, France). ST2-deficient mice (ST2^−/−^) on C57BL6/J background were generated and provided by Andrew N. J. McKenzie (Medical Research Council Laboratory of Molecular Biology, Cambridge, UK) (35).

### Treatments

Bleomycin sulfate (7.5 and 3 mg/kg for 1 and 11–14 days experiment, respectively; Bellon Laboratories) in saline or saline alone were given through the airways by nasal instillation in a volume of 40 µl under light ketamine–xylasine anesthesia. The mice were monitored daily.

### Bronchoalveolar Lavage (BAL) and Cell Counts

Mice were sacrificed, and BAL was performed as previously described (6). Differential cell counts were performed on cytospin preparation (Cytospin 3, Thermo Shandon) after May–Grünwald–Giemsa staining (Sigma-Aldrich, St. Louis, MO, USA) according to the manufacturer’s instructions. Differential cell counts were made on at least 200 cells using standard morphological criteria.

### Lung Homogenization and Analysis

After the BAL, the lungs were perfused with isoton to flush the vascular content. Lungs were homogenized by a rotor-stator (Ultra-turrax^®^) in 1 ml of PBS. The extract was centrifuged 10 min at 10,000 rpm, and the supernatant was stored at −80°C before mediator measurements or lung tissue MPO activity as described (36). The MPO activity was evaluated as described (6).

### Flow Cytometry Analysis

After perfusion, lungs were removed from mice and cut before being digested with DNase (Sigma, 1 mg/ml) and Liberase (Roche, 5 mg/ml) for 1 h at 37°C with stirring. After digestion, cells were filtered on a 40 µM filter, and red bloods cells were lysed with Lysing buffer (BD Pharm Lyse™—BD Pharmingen). Cells were incubated with antibodies (Table 1) during 20 min at 4°C in PBS, 2% FCS, 2 mM EDTA, and washed twice with PBS, 2% FCS, 2 mM EDTA, and fixed with lysing buffer 1 (BD Pharmingen). Data were acquired with a flow cytometer (BD FacsCanto II) and analyzed with Flow Jo.v7 software (Tree Star, Mountain View, CA, USA).

**Table 1:** List of antibodies used for flow cytometry.

Antibody	Clone	Fluorescence
Ly6G (Gr-1)	RB6-8C5	PeCy7
TCRb	H57 597	PeCy7
CD11b	M1/70	PerCP Cy5.5
B220	RA3-6B2	PerCP Cy5.5
Siglec F	E50-2440	PE
CD8a	53-6.7	PE
CD11c	HL3	FITC
CD3e	145-2C11	FITC
Live dead	–	APC-Cy7
CD206	MR6F3	APC
CD45	30-F11	APC
CD45	30-F11	V500
F4/80	BM8	V450
CD4	RM4-5	V450

### Lung Histology

The left lobe of lung was fixed in 4% buffered formaldehyde, processed, and paraffin embedded under standard conditions. Lung sections of 3 µm were stained with picrosirius red stain specific of collagen (Sigma-Aldrich). The slides were blindly examined by using a Leica microscope at 200× magnification (Leica, Solms, Germany). Inflammatory cell infiltration, edema, and interstitial fibrosis were assessed by a semi-quantitative score (with increasing severity 0–5) by two independent observers.

### Mediator Measurements

For cytokine determination, BALF supernatants and lung homogenates were analyzed by ELISA assay kits for murine IL-33, CXCL1/KC, CCL2/MCP-1, CCL17/TARC, MMP-9, TIMP-1, and IL-6, according to the manufacturer’s instructions (R&D System, Minneapolis, MN, USA). In addition, CCL5/Rantes, IL-4, IL-5, IL-6, IL-10, IL-13, and IFN-γ were measured by multiplex immunoassay and revealed with MagPix reader (Bio-Rad) according to the manufacturer’s instructions. Data were analyzed with Bio-Plex Manager software (Bio-Rad).

### Total Lung Collagen Measurements

Lung homogenate aliquots (50 µl) were assayed for lung collagen levels using the Sircol collagen dye binding assay according to the manufacturer’s instructions (Biocolor Ltd., Northern Ireland).

### MRI Methodology

Magnetic resonance imaging was performed on a 9.4 T horizontal ultra-shielded superconducting magnet dedicated to small-animal imaging (94/20 USR Bruker Biospec, Wissembourg, France) equipped with a 950 mT/m gradient set. A 35-mm Bruker birdcage RF coil was used. The operational software for acquisition and analysis was Paravision PV5 (Bruker). During MRI acquisition, the mouse was placed in supine position in a cradle made of Plexiglas. The head is immobilized by means of a bar teeth. Following a short period of induction in a box, anesthesia was maintained with inhaled isoflurane 1.5–2% in a mixture of air/O_2_ (1:1) administered with a flow rate of 0.5 l/min *via* a nose mask. Body temperature was maintained at 37 ± 1°C using a warm-water circulation system. Breathing rate was controlled throughout the experiment using a pressure sensor placed on the abdomen. All images were performed by synchronizing the acquisition on the breath of the animal to suppress artifacts caused by movements of the chest. An intragate flash sequence with the following parameters was used throughout the study for the detection of BLM-induced lung injury in mice: repetition time 6.9 ms, echo time 3.727 ms, field of view 3 cm × 3 cm, matrix size 512*512, flip angle = 20°, bandwidth 75.7 kHz, slice thickness 570 µm. The total acquisition time was 2 min and 58 s for an axial slice. Three resolved images (59 μm × 59 μm × 570 μm) were recorded with an inter-slice distance of 1 mm. The first image is located in the bronchi, and the last in the bronchioles.

### MRI Image Analysis

The area of BLM-induced lesions was quantified on each image from the data set. A region of interest was drawn to manually segment the lungs. It is worthwhile to mention that this procedure includes vasculature in the segmentation since the signal from edema and vessels are of comparable intensities particularly in dosed animals. The background noise was measured from each image. Results were expressed as the ratio of the intensity of pixels in the lungs/background noise on each image. The evolution of the intensity of lungs level signal was then studied at the same time point, before (day 0) and after instillation of NaCl or BLM (days 1, 7, and 14) in control or ST2^−/−^ mice.

### Statistical Tests

Statistical analysis for different groups was done using the parametric one-way ANOVA with Bonferroni’s multiple comparison test. The results were considered significant at *p* < 0.05.

## Ethics Statement

All animal experimental protocols complied with the French ethical and animal experiments regulations (see Charte Nationale, Code Rural R 214-122, 214-124 and European Union Directive 86/609/EEC) and were approved by the “Ethics Committee for Animal Experimentation of CNRS Campus Orleans” (CCO), registered by the French National Committee of Ethical Reflexion for Animal Experimentation, under No. CLE CCO 2015-1087.

## Author Contributions

NR made substantial contributions in the analysis and interpretation of data, made the figures, and participated in writing the manuscript. MF made substantial contributions in the conception and design of the study and acquisition of data and participated in writing the manuscript. IM, LB, CS, MA, MLB, MN, and AG made substantial contributions in the conception and design of the study and acquisition of data. VQ and BR made critical revisions of the manuscript. SM and WM made substantial contributions in the conception and design of the study and acquisition of data, in the analysis and interpretation of data and participated in writing the manuscript. IC made substantial contributions in the conception and design of the study and acquisition of data, in the analysis and interpretation of data and wrote the manuscript.

## Conflict of Interest Statement

The authors declare that the research was conducted in the absence of any commercial or financial relationships that could be construed as a potential conflict of interest.

## Supplementary Material

Figure S1 Gating strategy of lung cells by FASC analysis. Gating strategy for flow cytometry analysis of cell subsets present in lung parenchyma at day 11 after bleomycin administration was shown. **(A)** Gating of hematopoietic cells (CD45^+^), living cells (Live-Dead), neutrophils (Gr1^high^), macrophages (F4/80^+^ CD11b^+^), M1 macrophages (CD206), and alternative macrophages or M2 macrophages (CD206^+^) was shown. **(B)** Gating of hematopoietic cells (CD45^+^), living cells (Live-Dead), B cells (B220^+^), and T cells (CD3ε^+^) is represented.

## Abbreviations

ST2, suppression of tumorigenicity 2; IL-33, interleukin-33; BLM, bleomycin; Th2, T helper type 2; sST2, soluble ST2.
