# Supplementary figures and images for: The IL-33 Receptor ST2 Regulates Pulmonary Inflammation and Fibrosis to Bleomycin
Source: Front Immunol. 2018 Jun 25;9:1476. doi: 10.3389/fimmu.2018.01476 (PMC6026799; doi:10.3389/fimmu.2018.01476)

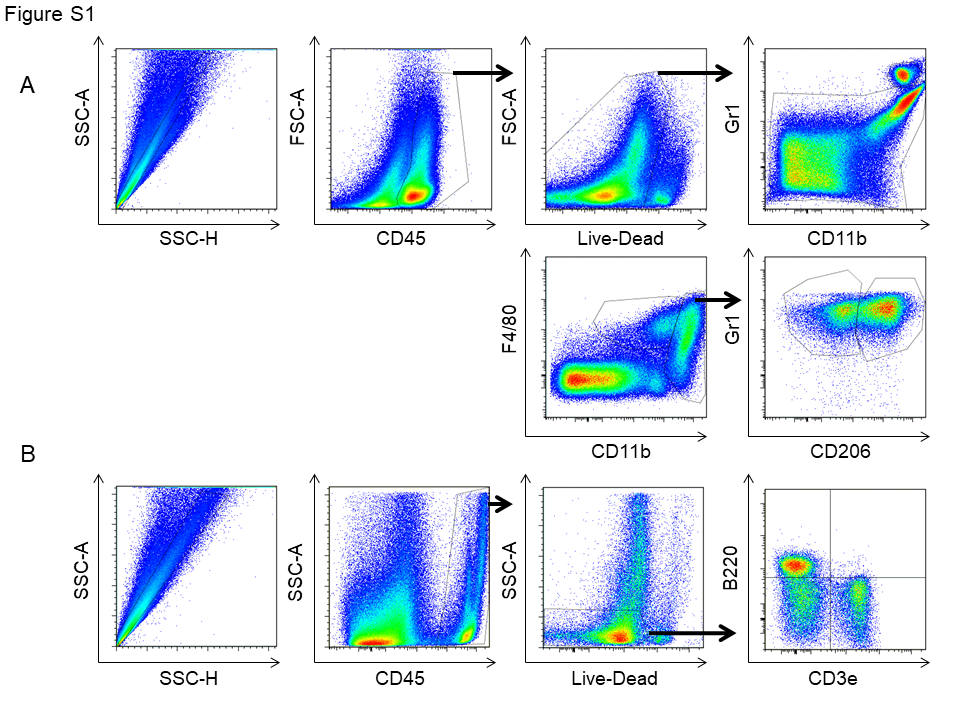

Supplement: Figure S1 — Gating strategy of lung cells by FASC analysis. Gating strategy for flow cytometry analysis of cell subsets present in lung parenchyma at day 11 after bleomycin administration was shown. (A) Gating of hematopoietic cells (CD45+), living cells (Live-Dead), neutrophils (Gr1high), macrophages (F4/80+ CD11b+), M1 macrophages (CD206), and alternative macrophages or M2 macrophages (CD206+) was shown. (B) Gating of hematopoietic cells (CD45+), living cells (Live-Dead), B cells (B220+), and T cells (CD3ε+) is represented. [file Image_1.tif]
